# Answer to an Unprovoked Attack

**Published:** 1884-07

**Authors:** 


### Editorial, Etc.

Answer to an Unprovoked Attack.—In the June No. of the *Independent Practitioner* appeared an article by Dr. B. Merrill Hopkinson, a graduate of the Baltimore College of Dental Surgery, in which he charged the present faculty of his *alma mater* with having, at its last commencement, graduated a number of gentlemen without requiring of them attendance upon the lectures, demonstrations, etc. In the July No. of the same Journal appeared a "reply" to this article of Dr. Hopkinson, signed by the faculty of the Baltimore College, which consisted of an altogether unprovoked attack upon the Dental Department of the University of Maryland, on the ground that Dr. Hopkinson is a member of the faculty of the Dental Department, and that the facts he stated could only have been obtained from the Dean of the latter institution. To this attack, the Dean of the Dental Department of the University of Maryland replies in a statement made under oath, and which will appear in the *Independent Practitioner* for August 1884. Dr. Gorgas in his answer to the attack, states in the first place, that Dr. Hopkinson is not connected with the Dental Department of the University of Maryland, having resigned his position as Demonstrator before the article in question was written, and subsequently to the publication of the annual Catalogue of 1884. Also that the faculty of the Dental Department of the University of Maryland had nothing whatever to do with Dr. Hopkinson's article, for which he alone is responsible. The "reply," of the faculty of the Baltimore College alleged that Messrs. Meeker and Barlow (two of the cases referred to by Dr. Hopkinson,) were guaranteed diplomas and promised easy examinations by Dr. Gorgas. Such a statement Dr. Gorgas denies most positively, although he was repeatedly urged by the two gentlemen to secure for them easy examinations, and asserts that he has never, during a period of of more than a quarter of a century of connection with dental schools, guaranteed a student a diploma or promised easy examinations. Dr. Gorgas in his answer also asserts that, owing to eighteen or twenty years of practice, reputation as dental practitioners, and home business, Messrs. Meeker, Barlow and Baldwin, who applied to Dr. Gorgas (the first two having matriculated at the Dental Department of the University of Maryland and attended some of its lectures, besides dissecting, &c.,) an arrangement was made with these gentlemen, by which during two sessions, they could meet the requirements of one session. Also that the only reason why these gentlemen went to the Baltimore College was that by doing so they could obtain a diploma without passing more than a day or two at that institution, whereas if they had continued at, and attended the University of Maryland some three months attendance at least would have been required of them. As regards another case, that of T. H. Schaeffer, also referred to by Dr. Hopkinson, the faculty of the Baltimore College alleged that Mr. Schaeffer (who had been out of practice for some eight years, and who applied to Dr Gorgas for a diploma on *no* attendance,) when he approached Dr. Gorgas, supposed him to be the Dean of the Baltimore College. Dr. Gorgas in his answer to this statement, positively asserts that Mr. Schaeffer made no mistake whatever, as he stated that he had been told that the diploma of the Dental Department of the University of Maryland was worth a great deal more than that of the Baltimore College. Dr. Gorgas also asserts that he refused point blank to permit Mr. Schaeffer to enter the University at the end of the session, and informed him that a full session would be required of him. Also that Mr. Schaeffer stated in one of the dental depots of Baltimore shortly after his interview with Dr. Gorgas, that he had been refused, but that he could obtain a diploma from the Baltimore College, as the Dean of that school had told him he could return home and come again to the Baltimore College at the end of the session for an examination. Dr. Gorgas asks what would be the nature of such an examination, when the appliant had been out of practice for some eight years, engaged in the manufacture of agricultural implements, and had never applied himself to the study of dental science? In regard to the cases of Messrs Dowsley and Twitchell, also referred to by Dr. Hopkinson, and who, after being at the Baltimore College for some days, and declaring themselves dissatisfied with that school, matriculated at the University, and a few days afterwards returned to the Baltimore College—Dr. Gorgas, judging from the letter of these gentlemen is of the opinion that such a return was occasioned by his refusal to credit these gentlemen with certain examinations they had passed as juniors at the Boston Dental College, for they informed him that they had made more satisfactory arrangements with the Dean of the Baltimore College.

In regard to a charge made in the reply of the faculty of the Baltimore College, that one of their students had been offered easy examinations and been guaranteed a diploma by Prof. James H. Harris, this gentlemen positively denies ever having made such an offer to anyone, and declaims having ever had the power to do so. The name of the student in question, however, is not given in the reply of the faculty of the Baltimore College. The above is a mere abstract from the answer of Dr. Gorgas to the altogether uncalled for attact of the faculty of the Baltimore College on the Dental Department of the University of Maryland.

